# Targeted therapies and immunotherapy in non-small-cell lung cancer

**DOI:** 10.3332/ecancer.2016.648

**Published:** 2016-06-23

**Authors:** D Cortinovis, M Abbate, P Bidoli, S Capici, S Canova

**Affiliations:** Medical Oncology Unit, AOU San Gerardo, via Giambattista Pergolesi 33, 20900 Monza, Italy.

**Keywords:** non-small cell lung cancer, targeted agents, oncogene drivers, immunotherapy

## Abstract

Non-small-cell lung cancer is still considered a difficult disease to manage because of its aggressiveness and resistance to common therapies. Chemotherapy remains the gold standard in nearly 80% of lung cancers, but clinical outcomes are discouraging, and the impact on median overall survival (OS) barely reaches 12 months.

At the end of the last century, the discovery of oncogene-driven tumours completely changed the therapeutic landscape in lung cancers, harbouring specific gene mutations/translocations. Epidermal growth factors receptor (EGFR) common mutations first and anaplastic lymphoma kinase (ALK) translocations later led new insights in lung cancer biology knowledge. The use of specific tyrosine kinases inhibitors overturned the biological behaviour of EGFR mutation positive tumours and became a preclinical model to understand the heterogeneity of lung cancers and the mechanisms of drug resistance. In this review, we summarise the employment of targeted agents against the most representative biomolecular alterations and provide some criticisms of the therapeutic strategies.

## Introduction

Lung cancer is still considered an aggressive disease and worldwide as a big-killer disease. The discovery of some oncogenes that drive the tumour growth will transform 10–20% of non-small-cell lung cancers to a more indolent disease. In clinical practice, EGFR sensitive mutations and ALK translocations change dramatically the therapeutic strategy with the introduction of TKIs instead of chemotherapy to manage tumour progression and metastatic spread attitude with an improvement of symptom controls and patient’s quality of life (QoL). Many more rarest mutations/translocations have been discovered recently, and the future therapeutic scenario seems to have further changed in the management of these diseases. Another breakthrough regards the ‘immunomodulation’ and the knowledge that this cancer is quite immunogenic as a way to control non-smal-cell lung cancer. It is thought that empowering the host immune system by check-point immunomodulatory agents could be a new promising weapon to disease control.

In this review, we report some insights about the present and near future management of oncogene addicted non-small-cell lung cancers with particular attention to practical clinical aspects; some points of view regarding PD1 and PDL1 Abs will also be discussed.

## EGFR-driven lung cancers

The EGFR pathway represents the pioneer of personalised medicine in lung cancer. The two most common activating mutations involve exon 19 and exon 21. In the first exon, there are in-frame deletions, while in exon 21 there is an amino acid substitution of leucine to arginine at codon 858 (L858R) [1, 2]. These mutations account together for more than 90% of activating EGFR mutations and are present in about 10% of Caucasian patients with non-small-cell lung cancer (NSCLC) and about 50% of Asian patients with NSCLC [1].

All the approved EGFR tyrosine kinase inhibitors (TKI) including gefitinib, erlotinib, and afatinib, demonstrated a statistically significant improvement of progression-free survival (PFS) compared to chemotherapy in EGFR selected patients with median PFS around 10–13 months. Despite this benefit, clinical trials did not manage to achieve a significant increase of overall survival (OS) [3–5]. This has been ascribed to the high rates of crossover to the targeted agent after progression with first line chemotherapy.

However, a combined analysis of LUX-Lung 3 and LUX-Lung 6 demonstrated a statistically and clinically significant OS benefit with afatinib compared to chemotherapy in patients with exon 19 deletions (31.7 versus 20.7 months; HR 0.59, 95% CI 0.45–0.77, p = 0.0001), whereas there was no difference in OS in patients with EGFR exon 21 mutations [6].

Furthermore, Kuan and colleagues carried out a meta-analysis of randomised controlled trials comparing EGFR TKIs with chemotherapy. They confirmed a PFS benefit for all the TKIs, but they evidenced a statistically significant OS benefit only in patients with exon 19 deletions, treated with irreversible TKIs [7].

**Figure 1.**
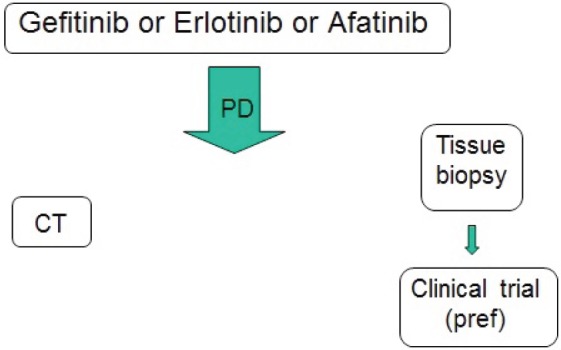
Actual therapeutic strategy for EGFR-positive advanced NSCLC.

Additional data derive from the LUX-Lung 7 trial. This is a phase IIb head-to-head trial which evaluated afatinib (40mg daily) versus gefitinib as first-line treatment of patients with EGFR-mutated lung adenocarcinoma (Figure 1). Afatinib significantly improved overall response rate (ORR) (70% versus 56%, p = 0.0083) and significantly reduced the risk of progression by 27% compared to gefitinib with the benefits growing over treatment period. In fact, a significant higher proportion of patients were alive and progression-free at 18 months (27% versus 15%, p = 0.018) and 24 months (18% versus 8%, p = 0.018). OS data are not yet mature. In addition, this study confirmed a different toxicity profile between the two drugs [8].

These data highlight the difference between reversible and irreversible EGFR TKIs, and confirm the different clinical behaviour and different sensitivity to TKIs between EGFR deletion 19 and L858R positive mutations lung cancer [9, 10].

A different sensitivity to EGFR TKIs is also present between lung cancer patients with common and uncommon mutations. Clinical benefit is evident, but lower in patients with uncommon mutations [11].

The most frequent rare mutations are G719X and L861G in exon 18, S768I in exon 20, and exon 20 insertions. The exon 18 mutations here reported are associated with a benefit from EGFR TKIs treatment, even if inferior to the benefit achieved in common mutations. The S768I is probably considered a mutation of partial resistance to EGFR TKI, while exon 20 insertions identifies patients refractory to EGFR TKIs and those who should receive chemotherapy. Uncommon mutations are more frequent in solid and papillary histologies [11, 12].

Therefore, patients with common mutations should receive EGFR TKIs as first-line treatment, whereas patients with rare mutations may be treated with targeted agent also in second or third-line because of the lower ORR and shorter PFS.

The choice of which TKI to employ should be based on the different toxicity profile, but patients with EGFR deletion 19 should be preferably treated with afatinib because of the available data on OS.

### Combination strategies to overcome resistance

Nowadays, the major challenge for these patients remains the onset of resistance to EGFR TKIs.

The timing of starting a new treatment depends on the speed and sites of evolution.

In fact, patients with EGFR-mutated non-small-cell lung cancer (NSCLC) experience a rapid progression of disease within three weeks after withdrawal of gefitinib or erlotinib [13]. Therefore, patients with central nervous system progression could carry on receiving the EGFR TKI with local treatment (surgery or radiotherapy) at the new site because of the suboptimal concentration of the drug in the brain.

Continuing an EGFR TKI beyond progression could be a promising strategy in selected patients, especially those with oligo-metastatic progressive disease to prolong PFS and to delay subsequent treatments further, eventually with the addition of local therapy [14, 15]. However, this approach should be evaluated in prospective randomised trials.

On the other side, when a fast and systemic progression occurs, a change of treatment strategy should be considered.

Clinicians made many efforts to overcome resistance, initially combining treatments with different mechanisms of action.

The combination of erlotinib and bevacizumab significantly prolonged PFS (16 versus 9.7 months, p = 0.0015) in patients with NSCLC with EGFR-activating mutations compared to erlotinib alone [16]. A phase III study is warranted to evaluate efficacy of this combination in a larger population.

Another treatment strategy to further delay progression is the combination of an EGFR TKI with chemotherapy. Sequential administration of EGFR TKIs and chemotherapy demonstrated greater efficacy than concurrent administration [17], and supported the development of clinical trials.

Based on the results of a phase II trial that demonstrated an advantage of PFS in Asian patients treated with a sequential administration of erlotinib and chemotherapy (gemcitabine plus cisplatin or carboplatin) [18], a phase III trial was conducted in the same population with primary endpoint PFS. This trial demonstrated a statistically significant improvement of both median PFS (16.8 months versus 6.9 months, HR 0.25 [0.16–0.39]; p < 0.0001) and median OS (31.4 versus 20.6 months, HR 0.48 [0.27–0.84]; p = 0.0092), in patients with EGFR activating mutations treated with chemotherapy plus erlotinib followed by erlotinib until progression, compared to those treated with chemotherapy plus placebo followed by erlotinib until progression [19].

Another strategy of association between EGFR TKIs and chemotherapy tested the possibility to continue the TKI with the addition of chemotherapy after disease progression in patients with EGFR-mutated NSCLC. A phase III multicentre study randomised patients to receive chemotherapy with cisplatin and pemetrexed plus gefitinib or chemotherapy plus placebo after progression to first line gefitinib. Unfortunately, this study did not meet its primary endpoint of improved PFS. Median PFS was 5.4 months for both groups (HR 0.86; p = 0.273) [20].

Another phase III trial evaluated afatinib plus paclitaxel compared to chemotherapy alone in patients with acquired resistance to erlotinib/gefitinib and afatinib. Afatinib plus paclitaxel significantly improved PFS and ORR compared to chemotherapy, but it did not improve OS. In this study, patients were unselected for EGFR mutation status [21].

### Second-generation EGFR TKIs

Second-generation EGFR TKIs captured interest because of their theoretical advantages making them potentially able to overcome resistance to first-generation EGFR TKIs. In fact, they have higher affinity for and irreversible binding to the EGFR kinase domain, they are pan-HER inhibitors, and they have *in-vitro* activity against T790-positive NSCLC cell lines [22].

Despite these premises, clinical studies were quite disappointing.

Afatinib was evaluated compared to placebo in a phase IIb/III trial in patients pretreated with one or two chemotherapy regimens and who progressed to gefitinib or erlotinib. Despite longer median PFS in the afatinib group than in the placebo group (3.3 months, 95% CI 2.79–4.40 versus 1.1 months, 0.95–1.68; HR 0.38, 95% CI 0.32–0.48; p < 0.0001), this study did not meet its primary endpoint of improved OS. Median OS was 10.8 months (95% CI 10.0–12.0) in the afatinib group versus 12.0 months (10.2–14.3) in the placebo group (HR 1.08, 95% CI 0.86–1.35; p = 0.74) [23].

Therefore, afatinib remains a good option in EGFR-mutant patients, naïve to EGFR TKIs.

Dacomitinib is another irreversible pan-HER TKI. In patients pretreated with chemotherapy and erlotinib or gefitinib, dacomitinib compared to placebo did not increase OS neither in patients with EGFR-mutation-positive tumours (HR 0.98, 95% CI 0.67–1.44) nor in patients with EGFR wild-type tumours (HR 0.93, 0.71–1.21; p_interaction_ = 0.69) [24].

Additionally, dacomitinib was investigated in a head-to-head phase 3 trial compared to erlotinib in patients pretreated with chemotherapy. Dacomitinib was not superior to erlotinib in an unselected patient population. Median PFS was 2.6 months (95%CI 1.9–2.9) in both groups (HR 0.941, 95% CI 0.802–1.104, p = 0.229) [25]. However, a pooled subset analysis from two randomised trials evidenced an advantage for dacomitinib over erlotinib, even if not statistically significant in EGFR mutation positive tumours [26].

Based on these data, a phase III trial comparing dacomitinib to gefitinib in first-line patients with EGFR-activating mutations is ongoing (ARCHER 1050).

However, awaiting these results, clinicians should consider higher incidence of adverse events, mostly diarrhoea, rash, and mucositis, associated with second-generation EGFR TKIs, probably because of inhibition of wild-type EGFR.

### Third-generation EGFR TKIs

The most promising drugs to delay progression are the third generation EGFR TKIs.

The discovery of mechanisms of acquired resistance to EGFR TKIs led to the development of these targeted agents. In fact, the most common mechanism of resistance to first-generation EGFR TKIs is the onset of the T790M mutation in exon 20 of EGFR. This secondary mutation accounts for about 50–60% of cases of acquired resistance and results in the substitution of methionine for threonine at position 790 in the kinase domain [27].

Osimertinib (AZD9291) and rociletinib (CO-1686) are the most advanced drugs in clinical development.

The first one was investigated in a phase I trial in patients with EGFR-positive NSCLC pretreated with EGFR TKIs and with radiologically documented disease progression. Primary objectives were safety, pharmacokinetics, and efficacy. A total of 253 patients were enrolled. No dose-limiting toxicities occurred. The most common adverse events were diarrhoea, rash, nausea, and decreased appetite. The ORR was 51% (95% CI 45–58). The median PFS was 9.6 months (95% CI 8.3–not reached) in the EGFR T790M-positive patients compared to 2.8 months (95% CI 2.1–4.3) in the EGFR T790M-negative patients [28].

Rociletinib (CO-1686) was evaluated in a phase I–II trial in patients with EGFR-mutated NSCLC who progressed to an EGFR TKI. Study objectives were safety, pharmacokinetics, and preliminary antitumour activity. 130 patients were treated. The ORR was 59% (95% CI 45–73) with a disease control rate (DCR) of 93% in EGFR T790M-positive patients and 29% (95% CI 8–51) with a DCR of 59% in EGFR T790M-negative patients. The median PFS was 13.1 months (95% CI 5.4–13.1) in EGFR T790M-positive patients and 5.6 months (95% CI 1.3-not reached) in EGFR T790M-negative patients. Hyperglycaemia was the most frequent toxicity [29].

It is interesting to note that rociletinib showed a good activity also in T790M negative tumours. Possible explanations lie in tumour heterogeneity, issues related to assay sensitivity, and direct activity of metabolites of rociletinib like IGF-1R (insulin like growth factor-1 receptor).

The use of third-generation EGFR TKIs in first-line obviously has a rationale to avoid the development of T790M mutation and delay time to progression. Phase III trials comparing AZD9291 (FLAURA, NCT02296125) and CO-1686 (TIGER 1, NCT02186301) with gefitinib or erlotinib in treatment-naïve patients with EGFR-mutation positive NSCLC are ongoing and recruiting.

Additional studies have already provided insights into the different mechanisms of acquired resistance to third-generation EGFR TKIs [30]. The comprehension of activity in T790M-negative tumours and mechanisms of resistance to third-generation EGFR TKIs are warranted for future investigations. These data could also be useful to establish the proper sequence strategy of EGFR TKIs.

Furthermore, interesting data may arise from the study of these drugs in early settings. Indeed, osimertinib is currently under evaluation as adjuvant treatment in resected NSCLC patients (AUDARA trial).

### Other mechanisms of resistance and promising strategies

Other mechanisms of acquired resistance include amplification of the gene encoding MET, PIK3CA mutations, BRAF mutations, ERBB2 amplification, and histologic transformation into small-cell lung cancer or epithelial mesenchymal transition [27].

The knowledge of these less frequent mechanisms of resistance is challenging and essential to better understand this disease and properly choose treatment strategies at time of progression to EGFR TKIs.

In the era of immune checkpoint inhibitors, the combination of anti PD1 antibody with EGFR TKIs should be evaluated as a potential strategy to delay resistance and improve survival.

In fact, Akbay *et al.* demonstrated that the activation of EGFR pathway increases PD-L1 expression. In particular, mice with EGFR-driven tumours show elevated PD-L1 expression. Furthermore, PD-1 blockade-induced tumour reduction and significantly increased OS in EGFR-mutant mouse models. In EGFR-mutant cell lines treatment with gefitinib-induced reduction of PD-L1 protein [31].

Similarly, D’Incecco *et al.* observed a strong correlation between PDL1 expression and presence of EGFR mutations in patients with NSCLC, and a significantly longer TTP in EGFR-mutated patients treated with gefitinib or erlotinib with PD-L1 positive disease than in PD-L1 negative disease [32].

Therefore, the combination of PD-1 blockade and EGFR TKIs warrants further studies as a promising strategy to delay resistance. Ongoing trials are assessing EGFR TKIs in combination with immune checkpoints inhibitors. Specifically, a phase I study is evaluating the combination of afatinib with pembrolizumab in patients with EGFR-mutant NSCLC progressing after a prior EGFR TKI (NCT02364609). A phase III trial is evaluating the combination of the PD-L1 inhibitor MEDI4736 with AZD9291 compared to AZD9291 alone in patients with T790M-positive NSCLC following a prior EGFR TKI (NCT02454933).

### Tissue versus liquid biopsy

Nowadays, a better understanding of the mechanisms of acquired resistance can be gatthered by making passes throughout, obtaining new tissue each time of disease progression. Clinicians should deal with this issue, not only in clinical trials, but also in daily practice to decide the further treatment strategy.

However, repeating a tissue biopsy is not always feasible and safe in patients with NSCLC. Furthermore, the tissue is not representative of the whole tumour because of the molecular heterogeneity of the disease.

Therefore, the employment of new techniques is necessary to limit invasive procedures, in addition to optimise assay sensitivity and reduce false positive results.

Next-generation sequencing (NGS) is widely used worldwide. It is able to discover all types of genomic alterations, including nucleotide substitutions, point mutations, insertions, deletions, copy number alterations, and chromosomal rearrangements. NGS may help to solve the problem of small biopsies containing few cells, the issues of low quality samples, and intra-tumour heterogeneity.

However, one of the major challenge of NGS remains to distinguish ‘driver’ mutations from ‘passenger’ events [33].

One of the main aims of new technologies is to obtain diagnosis and biomolecular characterisation from ever smaller samples, including blood samples.

Liquid biopsy consists of a blood-based test to identify tumour-associated genetic alterations. It can be used to assess circulating cell-free DNA (cfDNA), circulating tumour cells (CTCs), and exosomes. The advantages of liquid biopsies are that they are easily obtainable and repeatable, minimally invasive, and useful for many employments. In fact, they may be employed not only to diagnosis for the biomolecular characterisation of NSCLC, but also during treatment to assess response, and to detect resistance earlier than clinical or radiological progression. In EGFR-mutant NSCLC, liquid biopsies may allow to early detect acquired resistance to EGFR TKI and to identify changes of EGFR mutations. However, some drawbacks should be considered, such as the costs of these technologies, low count of CTCs in the blood, and the lack of standardised methods for isolation of CTCs [34]. Conversely, cfDNA is more easily detectable and is often present at high levels in blood. There are several platforms for the detection of EGFR mutations in cfDNA in NSCLC with different sensitivity [35].

Despite these obstacles, scientific researchers and cancer organisations should join their efforts to obtain ever more precise technologies to avoid false negative or false positive results and to overtake the problem of tumour heterogeneity with minimally invasive and easily repeatable approaches.

Preliminary clinical results are encouraging. Oxnard *et al* employed droplet digital PCR to assess response and emerging resistance to erlotinib in nine patients with EGFR-mutant NSCLC. They demonstrated high sensitivity and 100% specificity detecting pretreatment EGFR mutations, plasma response to erlotinib, and onset of plasma T790M before radiological progression [36].

Also, Karlovich and colleagues evidenced a good concordance of ORR between patients treated with rociletinib having T790M detected by plasma and those patients with T790M identified by tumour tests (44% versus 52%). Furthermore, plasma tests identified T790M mutations missed by tissue test, probably because of intra-tumour heterogeneity or inadequacy of tissue [37].

In conclusion, the best treatment for patients with EGFR-positive NSCLC is actually an EGFR TKI. The choice of which TKI to employ should be mainly based on the different toxicity profiles. However, clinicians should consider afatinib in patients with exon 19 deletion because of the available data on OS. Patients with uncommon mutations may be treated with chemotherapy in first-line, receiving a targeted agent in second-line.

**Figure 2.**
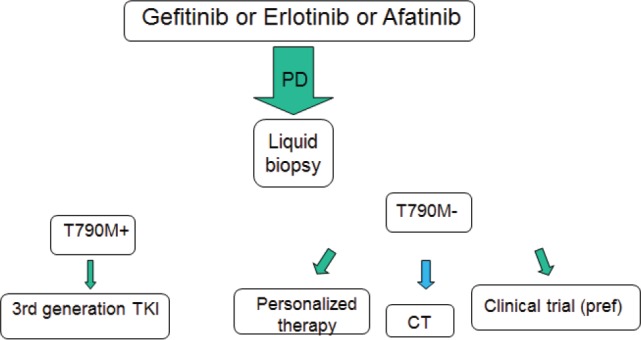
The next future therapeutic strategy for EGFR-positive advanced NSCLC.

**Figure 3.**
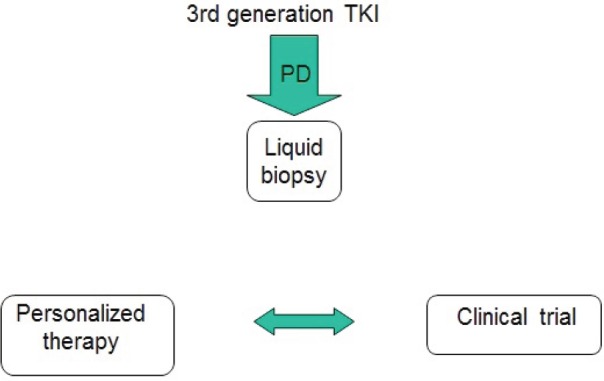
Late future therapeutic strategy for EGFR-positive advanced NSCLC.

At disease progression, different strategies should be evaluated, including continuing the EGFR TKI, according to speed and sites of evolution (Figure 2). The most promising drugs are the third-generation EGFR TKIs, currently available only in clinical trials in Europe. Interesting results may arise from ongoing first-line studies. Furthermore, their efficacy in metastatic patients warrants the evaluation in early stage disease. Today, a tissue or a liquid biopsy at disease progression is not mandatory in routine clinical practice, but it is useful to include patients in clinical trials. However, in the near future it will probably be necessary not only to better understand the acquired resistance mechanisms, but also to reach the goal of personalised medicine avoiding invasive approaches (Figure 3).

## ALK-driven lung cancers

### Introduction

The first evidence of differences between anaplastic lymphoma kinase (ALK) gene-echinoderm microtubule-associated protein-like 4 (EML4) translocated and wild-type counterparts lung tumours arose in 2007 [38].

It was evident that a small part of non-squamous lung cancers (4–6%) and very rare squamous lung cancers, probably with mixed histology, carried this gene fusion [39].

This discovery was important because it occurred along with the discovery that this rearrangement defined a group of lung tumours transformed and sustained in their growth by this translocation.

The ALK translocation with different genes defined the second biggest group of so called oncogene-addicted tumours after EGFR mutated ones, but this evidence happened with the discovery of activity *in vitro* cell lines and *in vivo* mouse models of ALK inhibitors [40, 41].

The ALK-translocated patients were identified as a category with different features from large lung cancer population. Generally these patients were younger, affected by adenocarcinoma showing sometimes signet-ring cells histology architecture, with a slight predominance of women, and the majority of them were former or never-smokers [42, 43].

Even though ALK translocations are generally mutually exclusive compared to EGFR and/or KRAS mutations, more recently the complexity of clonality, the resistance mechanisms, and the selective pressure of antiblastic treatments on tumour growth have made this principle more debatable [44–46].

Patients harbouring ALK translocations have a prognosis generally considered severe compared to that of its wild type counterpart; however, confounding data have emerged from the literature about the prognostic role of ALK translocation in lung cancers. In a cohort of NSCLC resected patients, ALK positivity seemed to be associated with poor prognosis [47]; however, the outcomes of patients treated with chemotherapy were similar to those observed in EGFR wild-type population [43]. It was also observed in retrospective series that in contrast, outcomes of EML4-ALK rearranged patients not treated with ALK inhibitor were better than for patients without translocation [48].

### How to detect ALK positivity

There are three major ways to detect positivity to ALK.

The first one to be employed was fluorescent *in situ* hybridisation (FISH); in particular, the Vysis ALK Break Apart FISH Probe Kit (Vysis FISH) (Abbott) is one of the most popular and accepted companion diagnostics [49].

This test displays some issues regarding the reproducibility of the results; also the outcomes are influenced by the readers’ expertise, the quality of tissue samples, and other aspects that are out of scope in this article.

In order to maximise the first step of screening, the currently used high immunohistochemistry (IHC) assay can be employed as well. One diagnostic test accepted around the world is VENTANA ALK (D5F3) CDx Assay. IHC is worldwide recognised as a reliable methodology with a good sensitivity that makes this test a stand-alone diagnostic tool for ALK TKI employment in clinical practice [50].

Finally, reverse transcriptase polymerase chain reaction (RT-PCR) is the more recent and more accurate way to study and detect ALK rearrangement and mutation. This procedure is usually performed in borderline FISH positive case, and it is useful to carefully study the pattern of resistance of secondary mutations arising after the exposure to first generation ALK inhibitor [51]. RT-PCR can simultaneously detect various ALK fusion partners; conversely, high quality RNA is required, and it can be difficult to obtain in routine clinical practice, particularly using standard FFPE tissue samples.

Amid the pros and cons linked to technical procedures for one to detect the ALK positivity, the main concern lies on the fact that on whom the test should be performed and when.

Nowadays, several experts’ consensus from AMP, CAP, and IASLC endorsed by ASCO stated that all patients with adenocarcinoma histology at advanced stage should be tested at the beginning of cancer diagnosis, regardless of their clinical characteristics, while testing is not recommended in non-adenocarcinoma histologies. However, if limited tissue is available and an adenocarcinoma component cannot be excluded, ALK testing may be performed even in squamous or small-cell histology. In these cases, clinical characteristics (i.e. never smokers and lung peripheral lesions) may be useful in selecting these patients for testing. Generally, primary tumours and metastatic lesions are equally suitable to determine ALK status for treatment selection.

Rebiopsy should be considered in clinical trials in order to detect the resistance mechanisms to first generation ALK inhibitors [52, 53].

### First generation TKI: crizotinib

Crizotinib is a first generation multi tyrosine kinases inhibitor of ALK, MET, and ROS1 that demonstrated a meaningful activity on ALK positive NSCLCs. In the earliest experiences with crizotinib in advanced and heavily pretreated NSCLC ALK positive patients, this drug showed an impressive response rate achieved in approximately 60% of patients with a median PFS of 7–10 months [39, 54, 55]. Given these promising results, crizotinib obtained US Food and Drug Administration (FDA) approval in 2011, specifically in ALK positive patients using the Vysis ALK Break-Apart FISH Probe Kit (Abbott Molecular, Inc.).

Consequently, crizotinib was compared to standard second line chemotherapy (docetaxel or pemetrexed) in a large phase III randomised trial. Crizotinib showed a superior mPFS (7.7 versus three months) without difference in terms of mOS (20.3 versus 22.8 months), probably because of the crossover effect [56].

Finally, in PROFILE 1014 trial, crizotinib was compared to the gold standard chemotherapy in first line setting (platinum + pemetrexed) with a significant difference in terms of the primary end point mPFS (10.9 versus 7.0 months). No difference was seen in terms of mOS because of the low rate of death from any cause at the time of data cutoff. The probability of one-year survival was 84% in the crizotinib group compared to 79% in the chemotherapy group [57]. The high crossover rate and the high sensitivity of ALK-positive tumours to pemetrexed containing chemotherapy accounted for this impressive performance of chemotherapy arm. The better outcome of pemetrexed in this population emerged also in the second line trial, in which patients treated with pemetrexed had a better outcome compared to docetaxel arm, even if inferior to crizotinib activity. More recently, we have been noting that low expression level of thymidylate synthase is associated with EML4-ALK fusion gene [58].

The analysis of updated results of phase I trial clearly showed that crizotinib is actually the best choice in patients carrying ALK positive tumours. The rate of patient ALK positive treated with crizotinib alive at one year was 70% compared to 44% in patients with the same molecular alteration who missed crizotinib therapy. This rate was similar to one-year survival of ALK wild-type population treated with chemotherapy [54].

Furthermore, there are consistent data across trials supporting a better safety of crizotinib compared to chemotherapy. The commonest adverse events like vision disorder, diarrhoea, vomiting, bradycardia, and transaminitis are generally low-grade, manageable, and reversible.

With a long-term follow up of published studies and real practice experience, some particular adverse events emerge, like hypogonadism, neutropaenia, and complex renal cysts [59]. All these newer adverse events, probably not fully understood and linked to multikinases inhibitory crizotinib activity, could be easily managed and monitored during normal therapy courses.

The astonishing activity observed from phase I–II studies as well as its good safety profile make crizotinib the first choice in chemo-naive or chemo-pretreated patients with ALK-positive NSCLC.

### Clinical and molecular pattern of resistance at a glance

Unfortunately, all patients treated with crizotinib experience disease progression after 8–12 months and a new therapeutic option should be considered.

There are two main perspectives to evaluate progression, a biomolecular and a clinical level.

From a biomolecular point of view, near one half of tumours ALK positive treated with crizotinib develop a secondary resistance mutation as gatekeeper mutation like L1196M, which gives a further tumoural ‘gain of function’ under the selective pressure of crizotinib exposure. Several other resistance mutations are observed, but a lot of them are important only academically. This resistance mechanism is also named ‘ALK-dominant’, which means that these kind of tumours maintain ALK as a gene drive to addition. Amplification of the ALK fusion gene is another ‘ALK-dominant’ strategy to escape ALK TKI resistance [60–62].

In the other 50% of cases, ALK loses its driver function and one or more bypass track mechanisms are responsible for resistance as activation or mutation of EGFR, KRAS, KIT, MET, and IGF-1R pathways or hypoxia-induced epithelial-mesenchymal transition (EMT). These patterns are recognised as ALK non-dominant resistance mechanisms [63–67].

All these biomolecular features of resistance are the core to understand the right and powerful strategy to treat patients after the failure of first hit inhibition on ALK positive tumours. However, in clinical practice these findings are rarely checked because of the lack of availability of accurate technical methodology or accessibility to appropriate drugs for different resistance mechanisms.

In the real world, clinical aspects remain the leading criteria to decide when to stop crizotinib and start a further line of therapy.

Generally, the pattern of progressive disease under crizotinib treatment leads the choice. One of the most recognised failures for crizotinib exposure was the development of brain metastases. In fact nearly 20–30% of patients present a brain involvement at diagnosis and one quarter of patients under crizotinib will develop CNS dissemination as the first and sole appearance of progressive disease. (This aspect is mainly because of a poor blood-brain-barrier penetration of crizotinib with several logarithm differences in term of plasma crizotinib ratio concentration between plasma and liquor [68].

An oligoprogressive disease noted as a slight progression in some or in all known lesions, or is an appearance of a new lesion in the brain, allows different clinical behaviours as described further in the text.

### From first generation to second generation ALK TKIs

Awaiting the second generation ALK TKIs, crizotinib is the only targeted agent to fight ALKpositive NSCLC with some differences to drug accessibility across countries. In particular in US crizotinib is licensed for first line treatment while in Europe crizotinib is indicated for the untreated ALK-positive patients, and in Italy the drug is reimbursed only in chemo-pretreated patients.

Clinicians learnt different lessons about the resistance and specific crizotinib weaknesses.

In particular, as the confidence in crizotinib use grew, some observations regarding the crizotinib employment beyond progression arose.

A retrospective analysis, conducted on 414 patients treated with crizotinib and enrolled in PROFILE 1001 and PROFILE 1005, showed a mOS benefit in the subgroup that continued crizotinib beyond progression, compared to those patients who stopped treatment when progressive disease was declared (16.4 versus 3.9 months) [69]. Even adjusting for confounding factors, crizotinib beyond progression is an effective strategy, in particular in the population who benefit from this drug and experience a slow progressive disease.

Another retrospective study showed that patients treated with first generation ALK TKI beyond progression had clinical benefits compared to the same patients treated with second generation ALK TKI. The main weakness of this trial is the inclusion of patients with slow oligoprogressive disease and those with faster progression, who have different biological and prognostic characteristics [70].

A second remarkable aspect is that the first main organ in which crizotinib show a weak control of metastatic spread is the brain. In fact, nearly 50% of patients treated with crizotinib experienced brain metastases as first failure site. This pitfall is because of the poor CNS penetration of crizotinib [71].

Usually, radiotherapy as stereotactic radiosurgery (SRS) technique or whole brain (WBRT) may be effective to treat patients that underwent progressive disease on brain during crizotinib use, especially without extracranial progression sites.

However, the mOS was significantly inferior in the population who initiated TKI before diagnosis of brain metastasis compared to those who started TKI after diagnosis of brain metastasis (28.4 versus 54.8 months) [72].

Overall, the mOS in this particular population is quite impressive (49.5 months), suggesting that the prognosis in patients with brain metastases and ALK-positive NSCLC should be completely revised compared to wild-type population [73, 74].

### Second generation ALK TKI: ceritinib, alectinib, brigatinib

More powerful agents, inhibiting preferentially ALK, have been discovered and already tested in clinical practice. Many of these drugs are under development in phase II and III trials in crizotinib refractory as well as in naive ALK positive patients.

Ceritinib (previously named LDK378) demonstrated activity against a wide range of secondary mutations, after the exposure to crizotinib in preclinical models and ROS1 positive and IGF1R positive patients [75].

In a phase I trial (ASCEND 1) [76], different doses of ceritinib were administered, from 50 mg to 750 mg once daily and mainly in ALK TKI pretreated patients. The maximum tolerated dose (MTD) was 750 mg with evidence of diarrhoea, vomiting, dehydration, transaminitis, and hypophosphataemia as major adverse events.

The overall response rate was 58%, with no difference between patients pretreated and not pretreated with crizotinib. The mPFS was seven months in the overall population; in the subgroup of pretreated patients, the mPFS was similar (6.9 months), while in naive patients an impressive mPFS of 10.4 months was reached. The clinical outcome of patients with CNS involvement was not different compared to patients without brain metastasis, and that intracranial responses were also observed in patients who developed progression at this site under crizotinib exposure.

Giving these striking results, ceritinib obtained a fast track approval by the Food Drug Administration (FDA) and more recently by Europena Medicine Agency (EMA), and it is now considered the first choice in patients with progressive disease after crizotinib use.

An update of ASCEND 1 trial [77] was performed and the activity of ceritinib was largely confirmed. Among 246 patients, the ORR was 58.5% with a slight difference in ALK TKI naive patients (66.3%). The mPFS was 8.2 and 6.9 months respectively in overall and pretreated ALK-TKI population. The mPFS period was not reached in ALK-TKI naive subgroup population with a PFS rate of 61.3% at 12 months. Again, an interesting activity in CNS disease was noted with an intracranial ORRs of 40% and 75% respectively in ALK pretreated and naive population.

Ceritinib development is ongoing with different phase II and III trials already closed to accrual or ongoing trials exploring the right setting of use of this compound in the therapeutic strategy: ceritinib in pre-treated patients > 1 platinum doublet and crizotinib (ASCEND 2), ceritinib in ALKi-naïve patients (ASCEND 3), ceritinib compared to chemotherapy in first line (ASCEND 4), ceritinib compared to chemotherapy in heavily pretreated patients (ASCEND 5), and ceritinib in patients with brain metastases (ASCEND 6).

Alectinib (RO5424802/CH5424802) is the second most studied compound after ceritinib.

It is a highly selective ALK inhibitor with activity also in RET translocated tumours.

The phase I/II study already published has been conducted in Japan [78] in ALK positive and ALK TKI naive patients. The dose of 300 mg b.i.d. was the recommended phase II dose. In the phase II trial, 43 out of 46 patients (93.5%) treated with alectinib had an objective response. These results forced the approval in Japan for advanced ALK positive lung cancer patients.

In a separate study in US, alectinib was evaluated in ALK rearranged NSCLC patients who progressed on or were intolerant to crizotinib. A total of 47 patients were enrolled at the doses ranging from 300 mg to 900 mg twice a day. In 44 patients assessed for activity, the ORR was 55% and 36% of them had stable disease. Among 21 patients with brain involvement at baseline, 52% obtained an objective response. With a good tolerability only two dose limiting toxicities (DLTs) occurred at 900 mg twice a day cohort and because of the optimal evidence of activity and tolerability and pharmacokinetic properties, the dose of 600 mg twice a day was selected for the phase II expansion cohort of the study [79].

In the phase II global study [80], alectinib at the dose of 600 mg taken twice a day showed an impressive activity in patients with advanced crizotinib-refractory ALK positive NSCLC, in particular in patients with CNS metastases. The ORR by independent review committee was 50% with a median duration of response of 11.2 months. The mPFS for all 138 patients was 8.9 months.

In the subgroup of 35 patients with CNS metastases at baseline, the ORR was 57% with a duration of response equivalent to that of patients without CNS involvement (10.3 months). Also in this study, alectinib showed a good tolerability profile with a few serious adverse events [80].

The phase II US and Canada study with alectinib in crizotinib ALK-positive resistant patients was recently published [81]. Around 69 (79%) patients out of 87 had measurable disease and were considered the response-evaluable population. Among them, 33 patients had a partial response confirmed by Independent Review Committee (IRC) and 22 patients obtained a stable disease with a disease control rate of 80%. The median duration of response was 13.5 months with an estimated PFS of 8.1 months. In the population with CNS involvement, 75% of patients achieved a prolonged objective response.

Thanks to these latter two phase II studies, the FDA approved alectinib for advanced ALK positive NSCLC patients resistant or intolerant to crizotinib [82].

Among other really promising second-generation ALK TKIs, brigatinib (AP26113) is worth mentioning because of its particular action. This compound demonstrated a peculiar activity also against ROS1, crizotinib-resistant mutations, and EGFR TKI-resistant mutations, including T790M resistance mutation [83].

An escalation phase I/II study was conducted on 57 patients [84]. The ORR was 69% in 51 crizotinib-pretreated patients and 100% of ALK TKI naive patients reached a response to brigatinib. Among 13 patients with a CNS involvement, 69% had an objective response. Given these promising results, this drug is studied in a phase II trial (ALTA) in advanced crizotinib-pretreated ALK positive NSCLC population.

### The third generation ALK TKIs and latest resistance model

There are many other ‘third generation’ ALK TKIs being currently studied and all these drugs have a different inhibition target compared to ALK fusion protein, IC50 blockade potency and activity on secondary mutations arising from first or second inhibition of ALK.

Among others, PF-06463922 (lorlatinib) has been studied in heavily pretreated crizotinib and ceritinib patients [85]. Studying the pattern of resistance to crizotinib or ceritinib, it clearly emerges that every ALK-positive tumours could virtually develop different secondary resistance mutations, which may be blocked by a second or third generation ALK-TKI. The mechanism that explain this situation is important; in fact, there are different subclonal ALK-positive malignant tumour cells (MTC) that contribute to ‘tumour mosaicism’. The employment of different ALK TKIs may differentially kill or select an emerging resistant clone that is responsible for progressive disease. It happens in primitive tumour as well as in all metastases resulting in different mechanisms to escape the blockade to ALK TKIs. However, drug selective pressure and stochastic generating mutations may resensitise tumours to previously employed ALK TKI.

Different biopsies, performed after progressing disease under crizotinib and lorlatinib use, explain the main resistance mechanisms responsible for progressive disease and could help to choose strategies to induce a new disease response.

In particular, in a clinical case reported by Shaw *et al* [85], a biopsy performed after crizotinib progression revealed a crizotinib-resistant ALK C1156Y mutation. This tumour was resistant also to ceritinib but was sensitive to lorlatinib. After further progression, a new biopsy showed a double-mutant subclone that maintained the C1156Y mutation and acquired L1198F mutation insensitive to lorlatinib. This situation resensitised tumour to further crizotinib employment with a radiologic response that lasted six months.

Recently the early results of a phase I/II study with lorlatinib in heavily pretreated chemo and ALK/ROS1 TKIs showed an interesting response rate of 44% with a safe profile demonstrating the activity of this compound even in tumours considered highly refractory to ALK inhibition [86].

### Hopes and criticisms about ALK TKIs employment

Undoubtedly, ALK TKIs are changing the quantity and the QoL of patients with ALK positive NSCLCs with a hope of mOS over two years. The new ALK TKIs, more potent and specific than crizotinib evaluated in ongoing clinical trials, could hold the promise to further improve the clinical outcomes.

The best strategy to treat these patients should be the use of a chemo-free therapeutic option at least in the first two lines of therapy, as suggested by scientific papers already published.

However, there is a disparity around the world about the access to the ALK TKIs in clinical practice. FDA and EMA, for example, licensed the use of crizotinib for first line treatment and ceritinib and alectinib (currently US FDA and Japan) for intolerant or resistant to crizotinib ALK-positive tumours. Nowadays in Italy, crizotinib is limited to second line setting after chemotherapy failure and second generation ALK TKIs are available only through a compassionate use programme. In other countries, economical issues or difficulty to perform ALK testing could hinder the access to the gold standard treatment for these patients.

The life expectancy of ALK-positive NSCLC patients will probably remain different across different countries.

The second main issue arises from the floor of resistance mechanism. There is a hiatus between the scientific development of second and third generation ALK TKIs and the indication done by regulatory agencies.

In particular, more than ten mutations have been identified to confer resistance to crizotinib and there is a different spectrum of ALK resistance mutations depending on the ALK inhibitor. Furthermore, what clearly emerges from preclinical models is that not all second and third generation ALK TKIs are equally active against every crizotinib resistant mutation.

For instance, alectinib is not active against the I1171T secondary mutations while ceritinib works; the opposite is true regarding the C1156Y and many other examples could be done [87]. The tumour resilience and the clonal evolution explain this ‘resistance mutations gene rating process’, and the scenario is more complicated taking into account also the non-ALK dominant escape phenomenon.

Ceritinib as well as alectinib and probably all the others compounds have been licensed in crizotinib-resistant patients, based on a clinical more than molecular definition of resistance.

This aspect will probably lead to give one compound of second and third generation after crizotinib resistance without any clinical activity; on the other hand this issue could be resolved performing biopsies (liquid or not) whenever progression occurs.

Another point regarding the best strategy is whether the new generation compounds should be employed at onset of crizotinib resistance or directly upfront.

Some trials, like ASCEND 4, are drawn to understand if ceritinib is superior to chemotherapy in naive ALK-positive NSCLC patients, while ALEX trial (NCT02075840) compares head-to-head alectinib and crizotinib in first line setting [88].

Waiting for clinical trial results, the second generation ALK TKIs must be employed after crizotinib failure. Some retrospective published experiences may guide clinicians to decide when to stop crizotinib for ceritinib or alectinib [70].

The right decisions should be probably based on different patterns of progression. In the oligoprogressive disease, crizotinib continuation plus loco-regional therapy (i.e. radiotherapy or surgery) is effective, while in the case of massive progression or deteriorating physical conditions switching to second ALK TKI is the best strategy.

Another issue regards the choice of the next-generation ALK inhibitors in clinical practice; at least eight compounds could be enter in clinical practice lacking randomised studies that compare next-generation ALK TKIs in the setting of crizotinib resistance.

The cross trial comparison is a risky exercise; however, the median duration of response, the CNS efficacy, and the tolerability profile showed by each next-generation ALK inhibitor in single-arm trials published so far will make the difference.

Finally, there is room for improvement in the management of ALK-positive NSCLC giving an ALK inhibitor in early phase of tumourigenesis.

The Adjuvant Lung Cancer Enrichment Marker Identification and Sequencing Trial (ALCHEMIST) [89] is the biggest trial ever designed in order to define the activity of crizotinib administered for two years compared to placebo in stage Ib (> 4 cm)-IIIA completely resected NSCLC (ALCHEMIST-ALK).

A positive activity of crizotinib in adjuvant setting will probably make the difference between a definitive cure of ALK positve NSCLC patients compared to a promise of a good ‘palliation’ seen so far.

### Practical insights in ALK-positive tumour management

Due to literature review and clinical trials scenario (Table 1) actually we suggest to test from the first diagnosis ALK in particular with IHC technique as screening methodology with further FISH analysis in case of doubts or mild IHC expression. All NSCLC, excepts squamous cell histology and never or former smokers must be tested.

**Figure 4.**
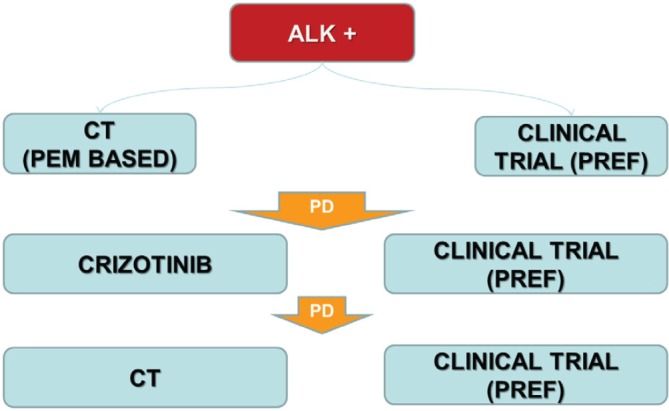
The therapeutic strategy of ALK-positive advanced NSCLC: current events in Italy.

In Italy chemotherapy with platinum/derivative + pemetrexed based regimens or clinical trial with next generation ALK TKIs (preferred option) should be used as first-line option (Figure 4). After progression to chemotherapy crizotinib is the gold standard treatment waiting for Italian Medicine Agency (AIFA) decision to license and reimburse the drug in first line setting.

The patients with an unequivocal clinical and/or image-guided progression should receive a second generation ALK TKIs on compassionate use programme or clinical trial (preferred option). The oligoprogressive disease (ie brain involvement or single extracranial metastasis) should be treated with loco-regional therapies continuing ALK TKI.

Probably the results from ongoing or recently concluded clinical trials will guide the clinical decisions about which of ALK TKIs could employ in first line or if there will be a preferred sequence from first and further ALK TKIs generation.

**Figure 5.**
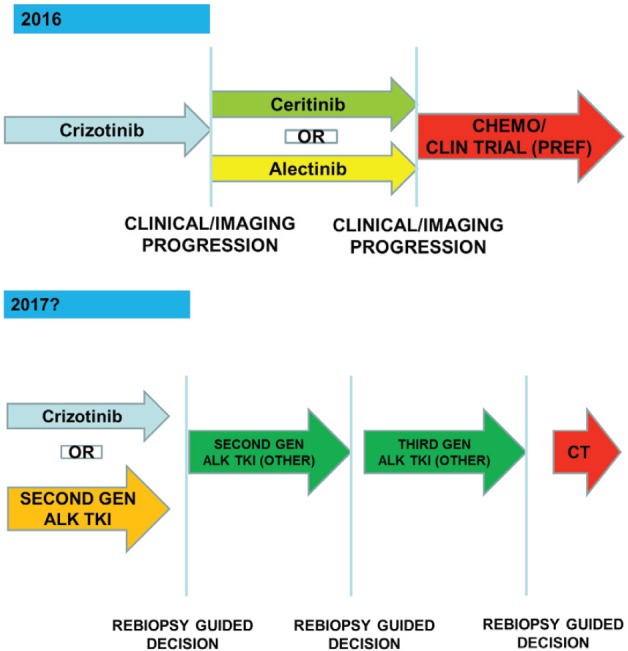
The optimal management of ALK-positive patients: dreaming the future.

Actually no clear role of re-biopsy emerges because of the complexity of the resistance mechanism to ALK TKI exposure. In the future, next generation sequencing techniques applied to tissue or plasma sample may guide clinicians to choose the right next generation ALK TKI to specific resistance mutation (Figure 5).

## Mutations or translocations other than EGFR/ALK

### BRAF

B-RAF mutations were identified in about 3% of lung cancers [90], these mutations activate the MAPK pathway [91]. The largest part of these mutations is V600E, and they are more frequent in current or former smokers.

There is evidence regarding B-RAF inhibition activity in lung cancer, such as that reported in some case reports [92, 93] and proved by interim analysis of a phase II trial, in which patients with BRAF V600E lung cancer beyond first line were treated with dabrafenib and obtained an ORR of 54% [94]. This trial showed a low ORR if compared to that attained with EGFR TKI (> 80%) and ALK TKI (> 60%), so to improve these results it should be useful to test the BRAF inhibitor with the MEK inhibitor.

Downstream inhibition using anti-MEK is a different way to inhibit the MAPK pathway.

An MEK1/MEK2 inhibitor, trametinib, combined with docetaxel was investigated with benefit in a phase I/Ib trial in lung cancer patients with wild type and mutated K-RAS [95].

A phase II single-arm non randomised trial using dabrafenib plus trametinib combination in Japanese patients with a confirmed BRAF V600E mutation in advanced NSCLC in any line of treatment is ongoing (NCT02672358). Primary end point is ORR whereas secondary end points are duration of response, PFS, OS, disease control rate.

### C-KIT

C-kit is a proto-oncogene that encodes a tyrosine kinase receptor (c-kit or CD117). It is related to the physiological development of some cellular line (haematopoiesis, spermatogenesis, melanogenesis) and to oncogenesis [96].

C-kit mutation is expressed in gastrointestinal stromal tumour (GIST) and it is a predictive biomarker of response to imatinib. C-kit overexpression is present in 40% of small cell lung cancer, and it seems to be correlated with poor prognosis [97], but it demonstrates the futility of target inhibition.

### HER-2

ERBB receptor is a family of tyrosine kinase receptors: EGFR (ERBB1), HER-2 (ERBB2), HER-3 (ERBB3), HER-4 (ERBB4). HER-2 is the preferred binding partner to other ERBB receptors.

HER-2 in NSCLC can be: a) overexpressed in 6–35% of cases, b) amplification in 10–20% and c) mutated in 1–4%.

#### HER-2 amplification and overexpression

HER-2 overexpression and amplification were studied in a phase II trial comparing weekly paclitaxel plus trastuzumab versus weekly docetaxel plus trastuzumab. In this trial, patients were stratified for HER-2 expression (HER-2 negative or HER-2 positive) in immunohistochemistry; in 52% of cases was made FISH. The trial was closed early for slow accrual; there were no differences between two arms for response rate and OS. HER-2 overexpression seems to be related to poor Performance Status [98].

In HER-2 positive patients (in IHC or FISH), treatment with cisplatin and gemcitabine versus the same chemotherapy plus trastuzumab showed the similar results for RR, TTP, and mPFS [99].

Actually, a phase II single-arm with trastuzumab emtansine in HER-2 IHC positive patients with locally advanced or metastatic disease after at least one prior chemotherapy regimen is ongoing. Patients were stratified in two separate cohort depending on IHC expression (2+ or 3+). Primary end point is ORR whereas PFS and OS are secondary end points.

#### HER-2 mutation

HER-2 mutation is more common in women, Asian people, adenocarcinoma, and never smokers.

Formation of HER-2/EGFR heterodimer justify the use of pan-HER inhibitors like afatinib, neratinib, and dacomitinib.

Five cases of NSCLC with HER-2 mutation and use of afaninib were reported; in three cases objective responses were registered [100].

Neratinib was tested in a phase II trial in NSCLC after erlotinib or gefitinib without benefit in the study population [101].

The preliminary data of dacomitinib in first-line treatment in NSCLC EGFR mutated patients or HER-2-mutated or -amplified showed 14% of partial response and 27% of stable disease in HER-2 mutated population [101].

HER-2 mutation is also the target of Hsp90 (heat shock protein 90). Ganetespib, Hsp90 inhibitor, prevents the binding of Hsp90 to co-chaperone p23. Some evidence of the benefit of HSP90 in NSCLC are demonstrated in mice models [101].

### JAK2

JAK 2 or *Janus kinase 2* is a gene responsible of making a protein that is involved in cell growth, proliferation, and in the JAK/STAT pathway. The JAK/STAT pathway is responsible for chemical signals transmission from outside the cell to the nucleus.

JAK mutation is rare in NSCLC and seems related to erlotinib acquired resistance. *In vitro* model of JAK2 inhibition and erlotinib given in lung cancer cell lines in combination with acquired erlotinib resistance seems to give back erlotinib sensitivity [102].

STAT3 can be constitutively activated in lung cancer. The first JAK inhibitor approved by FDA for haematologic disease is ruxolitinib, and the use of ruxolitinib in STAT3 activated lung cancer cell line does not prove activity [103]. Currently, clinical trials with ruxolitinib in NSCLC are ongoing.

### KRAS

KRAS (*Kirsten rat sarcoma viral oncogene homolog*) mutation is a common oncogenic driver observed in different solid cancers like colorectal cancer, pancreatic cancer, and lung cancer. KRAS sends signal from activated extracellular receptor to nucleus through RAS-RAF-MAPK and PI3K-AKT pathways.

KRAS mutation in lung cancer is about 26% in smoker patients and 6% in never smokers [104]. In general, the presence of KRAS mutation excludes EGFR mutation or ALK translocation. It is not druggable, so one way to inhibit KRAS is to use the inhibition of its downstream pathway, like MEK inhibition. A phase II trial in KRAS mutated lung cancer patients compared docetaxel alone to docetaxel with selumetinib (an anti-MEK1/MEK2). The combination showed an advantage for OS (5.2 months versus 9.4 months, HR 0·80, p = 0.21), PFS (2.1 m versus 5.3 months HR 0·58, p = 0·014) and ORR (0% versus 37%, p < 0·0001) [105]. A phase III trial is currently ongoing in KRAS mutated patient beyond first line: Selumetinib plus docetaxel versus placebo plus docetaxel with the primary end point being PFS [95, 105].

Mellema *et al* investigated platinum first line association with a second agent (pemetrexed, taxane, gemcitabine, bevacizumab plus taxane) in KRAS-mutated patients. It was proven that NSCLC KRAS-mutated patients had better ORR if treated with taxane [106].

Patients carrying KRAS mutation are often smokers, it is known that smoking cigarettes cause a lot of mutations and the presence of KRAS mutation is correlated with a high number of mutations. Among patients responder to PD-1 inhibitor, 50% carried KRAS mutation. Probably immunotherapy is a good therapeutic change in these patients [107].

### C-MET

*C-Met* is a proto-oncogene, and its product is a protein, the tyrosine kinase receptor c-MET. C-MET is normally expressed by epithelial cells, and it can be overexpressed by cancer cells.

Its ligand is hepatocyte growth factor (HGF), which provokes c-MET pathway activation and cells proliferation.

MET interacts with VEGF (Vascular Endothelial Growth Factor) promoting angiogenesis, so MET pathway can become one responsible of acquired resistance to VEGF pathway inhibitor [108]. Furthermore, C-MET amplification is described as a mechanism of non-T790M acquired resistance to anti-EGFR agent.

*Tivantinib* (ARQ197) is a small molecule that inhibits MET tyrosine kinase receptor in MET-expressing cancer cells inducing cells apoptosis. Phase II trial compared erlotinib plus tivantinib to erlotinib alone. The combination showed an advantage for PFS, but not for OS and ORR. A greater benefit in MET positive tumours was noted.

The combination of erlotinib and tivantinib versus erlotinib plus placebo was tested in non-squamous NSCLC phase III trial, beyond first line chemotherapy and EGFR TKI naive. The trial was discontinued for futility; it failed the primary end point OS. Subgroup analysis showed an advantage in combination arm for OS and PFS in patients with high MET expression [109].

*Onartuzumab* (Met Mab) is a monoclonal antibody targeting MET receptor. A phase II trial compared onartuzumab plus erlotinib to erlotinib alone in advanced NSCLC. The data suggests no improvement in ITT population for PFS and OS and an advantage for PFS and OS in C-MET positive NSCLC treated with combination [110]. The subsequent phase III trial was discontinued for futility [111]. MET evaluation was done on archival specimen, often on first biopsy, so MET values may not reflect the real status. Furthermore, the epitope targeted by onartuzumab was probably different from protein expression determined in IHC [112].

*Crizotinib* was synthetised as MET inhibitor, before discovering its dramatic response in ALK-positive NSCLC. Crizotinib was tested in 13 FISH MET amplified patients showing an antitumour activity and safety [113]. Crizotinib had also showed an activity in MET mutated tumours that represented 4% of adenocarcinoma patients in particular in 14 exon skipping mutation. In an ongoing study seven patients harbouring MET mutation were treated with crizotinib (three patients) and cabozantinib (three patients), there was partial response, only one patient had stable disease by response evaluation criteria in solid tumours (RECIST) criteria and complete PET response criteria in solid tumours (PERCIST) response [114].

*Cabozantinib* is an oral small tyrosine kinase inhibitor that targets VEGFR, MET, and RET. It was tested in a phase I trial in association with erlotinib showing safety and efficacy. Currently, phase II is ongoing.

### FGFR

FGFR (Fibroblast Growth Factor Receptor) is a tyrosine kinase receptor (FGFR1, FGFR2, FGFR3, FGFR4) responsible of PI3K/AKT and RAS/MAPK pathway activation. The binding of the receptor with its ligand supports tissue homeostasis like repair, inflammation, and angiogenesis, but FGFR deregulation because of genetic alteration can promote carcinogenesis. FGFR1 amplification seems more frequent in squamous NSCLC than in adenocarcinoma and seems related to cigarette smoking. FGFR inhibitor are cediranib, nintedanib, pazopanib, and ponatinib. *Cediranib* did not prove efficacy in an early randomised trial. *Nintedanib* plus docetaxel compared with docetaxel demonstrated efficacy in adenocarcinoma. *Pazopanib* (a VEGFR and FGFR inhibitor) provoked heavy toxicity. Trials with *ponatinib* are ongoing. A novel pan-FGFR inhibitor *BGJ398* in a phase I trial demonstrated safety and efficacy [115].

### PIK3CA

PI3K /AKT/mTOR (*phosphoinositide-3-kinase/v-akt murine thymoma viral oncogene homolog 1/mechanistic target of rapamycin*) pathway is often involved in various neoplasms because of dysregulated cascades signalling. Signal activation can start from cell surface (RTK-tyrosine kinase receptor, G-protein coupled receptor) or from cytoplasm through RAS protein. PI3K pathway oncogenic activation can occurs in several ways.

1. Mutation or amplification of gene encoding:
  - RTKs: EGFR, HER2
  - Subunits of PI3K: p110alfa, p110beta, p85alfa, and p85beta
  - AKT
2. Activating isoform of RAS
3. PTEN: mutations, deletions, loss of functions, or epigenetic silencing

PI3K pathway activation is responsible for cell growth, survival, and proliferation, and therefore this pathway takes a crucial role for anticancer strategy.

PI3K mutations were observed in approximately 4% of squamous cell carcinoma and 2.7% of adenocarcinoma. In adenocarcinoma lung cancer it was found that PI3K mutation can co-exist with EGFR mutation or KRAS mutation.

Multiple PI3K inhibitors are in development (GDC-0941, BKM120, XL147); several trials recently completed were leaded with PI3K inhibitor as single agent (NCT01501604) or in association to chemotherapy (NCT00974584, NCT00756847) or other agents (NCT00974584) [116].

### RET

RET rearrangement is the product from the fusion of RET gene and six partner genes, more frequent partners are *KIF5B* and *CCDC6* observed in approximately 1–2% of lung adenocarcinoma. RET rearrangement does not co-exist with EGFR mutation, ALK rearrangement, KRAS, BRAF, or HER-2 mutations. It was observed in women, young, never or former smokers, and adenocarcinoma histology. Several TKIs proved activity against RET kinase.

*Cabozantinib* is an oral small molecule multi-tyrosine kinase inhibitor and potent inhibitor of RET. Preliminary data of a phase II Memorial Sloan Kettering trial demonstrated an activity against RET rearranged cells. Currently, the trial is ongoing (NCT01639508) [117].

*Vandetanib* is an oral small molecule tyrosine kinase that target HER-2, VEGFR, EGFR, and RET. It was proven its activity in RET rearranged patient [118] and a phase II trial is ongoing (NCT01823068).

*Sunitinib* and *sorafenib* are TKIs that can target VEGFR and RET; they showed activity in *KIF5B*- *RET* rearranged NSCLC [119]. Furthermore, they are ongoing or recently closed trials with *lenvatinib* (NCT01877083) and *ponatinib* (NCT01813734).

*Alectinib* is an oral ALK inhibitor, that has demonstrated also an activity against RET rearrangement confirmed in mouse models. Alectinib proved activity also against RET mutations (KIF5B-RET V804L and V804 M) known as acquired resistance mechanism [120].

### ROS1

ROS1 fusion is the product of chromosomal rearrangement of ROS1 gene. It is identified in approximately 1% of lung cancers, and it is more common in never or light smokers and in adenocarcinoma histology. It is mutually exclusive with ALK. ROS1 receptor has a TK domain; its activation provokes cell growth and proliferation. Kinase receptor of ALK and ROS1 is similar, so they can be blocked with the same drugs.

In an expansion cohort of a phase I study using crizotinib, 50 ROS1 rearranged positive patients were treated with crizotinib 250 mg twice daily. It was observed an ORR of 72% and PFS of 19.2 months [121].

*PF-06463922* (lorlatinib) and* cabozantinib* showed activity in ROS1 positive, crizotinib-resistant cell lines [122, 123].

A phase II trial using *ceritinib* (LDK378) in ROS1 positive patients is ongoing (NCT02186821).

## Immunotherapy

PD-1, PDL-1

Immunotherapy uses drugs to stimulate the immune system versus cancer cells.

PD-1 (*programmed cell death 1*) is a protein expressed by activated immune cells, like T lymphocyte, B lymphocyte, natural killer (NK) cells, and macrophages. PD-1 has two ligands, PD-L1 (*programmed cell death 1 ligand 1*) and PD-L2 (*programmed cell death 1 ligand 2*).

PD-L1 is expressed by haematopoietic cells, endothelial cells, and by cancer cells; PD-L2 is expressed by antigen presenting cells.

PD-1 and PD-L1/PD-L2 interaction downregulates T cell activation and inhibits immune response.

It seems that tumour with high mutational burden is more sensitive to antiPD1 and anti PD-L1 agent.

Most described side effects are fatigue, rash, nausea, diarrhoea, infusion reaction, and decreased appetite. Less common are hypothyroidism and pneumonitis.

### PD-1 inhibitor

#### Pembrolizumab

Pembrolizumab is an antibody anti-PD1; it avoids inhibition of the immune system. A phase I trial evaluated pembrolizumab in metastatic NSCLC; all patients were evaluated for PD-L1 expression in a novel biopsy. The mPFS obtained in all patients with a PD-L1 expression of least 50% was 6.3 months, 6.1 months for pre-treated, and 12.5 months for untreated patients. The mOS of patients with strong PD-L1 expression (at least 50%) was not reached in the total population and in previously treated and untreated patients. When PD-L1 expression was weaker (proportion score of 1 to 49% or a score of less than 1% ), PFS and OS were shorter. The median duration of response was similar irrespective of PD-L1 expression [124]. So pembrolizumab has gained US FDA approval for PD-L1 > 50% NSCLC.

#### Nivolumab

Nivolumab is an antibody anti-PD1; it was evaluated for non-squamous and squamous NSCLC.

In Checkmate-057, phase III trial, non-squamous NSCLC patients were treated with nivolumab or docetaxel after first line therapy. Primary end point was OS. OS was 12.2 months for nivolumab arm and 9.4 months for docetaxel arm (HR 0.73, p = 0.002), RR was 19% versus 12% (p = 0.02), PFS was 2.3 months versus 4.2 months. Nivolumab seems more effective compared to high PD-L1 expression evaluated with IHC [125].

Checkmate-017 is a twin study in squamous NSCLC population. Nivolumab arm was significantly better for OS, PFS, and RR, and the benefit was independent from PD-L1 expression (OS 9.2 months versus 6.0 months, HR 0.59, p < 0.001; RR 20% versus 9%, p = 0.008, PFS 3.5 months verus 2.8 months, HR 0.62, p < 0.001) [126].

Nivolumab elicits fast and durable responses. A limit of these studies is the evaluation of PD-L1 expression, because it can change over time and these trials collected tumour specimen from archival or recent biopsy, so PD-L1 values may not reflect the real status.

Currently nivolumab has received approval in pretreated squamous and non-squamous NSCLC.

First line trials with nivolumab and pembrolizumab versus chemotherapy are ongoing.

### PD-L1 inhibitor

PD-L1 inhibitors were tested in phase I trials in NSCLC; they showed durability of response similar to PD-1 antibody, ORR of 14–24%, and stronger response compared to PD-L1 expression. The trial conducted with atezolizumab evaluated PD-L1 expression for tumour-infiltrating cells and cancer cells, and it was noticed that responses to therapy were correlated to PD-L1 expression in tumour- infiltrating cells.

#### Atezolizumab

Atezolizumab was compared to docetaxel in POPLAR, a randomised phase II trial. There was stratification for PD-L1 expression in tumour cells and tumour-infiltrating cells. It was noted an advantage in overall population treated with atezolizumab for OS, but not for PFS and RR. The real advantage was noted in stronger PD-L1 for OS, PFS, and RR [127].

#### Durvalumab and avelumab

These are monoclonal antibodies directed against PD-L1. They were accelerated into phase III clinical trial currently ongoing.

### Conclusions

Today, it is undebatable that testing the ‘mutational status’ of lung cancer could be useful in nearly all patients suffering from inoperable NSCLC, and it should be done at first diagnosis in order to put together the best therapeutic strategy. This analysis needs appropriate quantity and quality of tissue sample. Often, tissue biopsy or re-biopsy technique is difficult to do because secondary mutations arising or bypass track resistance occurrence even though we know that the knowledge of changing in mutational status after targeted agents exposure is essential.

Liquid biopsy is an innovative and the easiest way for test real-time mutational status situation. Recently, with next generation sequencing techniques (NGS), it is possible to test in parallel on plasma/serum circulating tumoural free DNA a thousand hot-spot gene alterations [128].

However, not all known mutations have approved drugs or are druggable. It is reasonable to say that more frequent and druggable mutations (for example EGFR, ALK, ROS1) must be done easily in real world practice, reserving rare mutations testing as screening during clinical trial management, in order to quickly address the patients carrying those rare mutations to more promising clinical trial.

Following a recent phase II randomised nationwide trial conducted in France, which enrolled heavy pretreated patients with solid tumour, it was clear there was danger to choose the therapy matching a molecular genetic alteration alone to a specific targeted agent.

Patients with a specific mutation were randomised to receive a selected target agent approved for a specific indication as experimental group or treatment at physician’s choice as control group. For all different mutations tested, the targeted agents employed had the similar mPFS compared to control [129]. Finally, the authors conclude that the use of targeted therapy should not be used off-label.

It is otherwise evident outside the clinical trials when a targeted agent is employed in clinical indication, for e.g. the results in term of activity and efficacy as demonstrated by The French Cooperative Thoracic Intergroup (IFCT) in a recent paper that outlines the effort of international guidelines applicability to clinical practice [130].

The evaluation of mutational status, beyond the most frequent ones is wishful. Patients carrying rare mutation should be addressed to clinical trials.

## Conclusion

Non-small-cell lung cancer is no longer considered a monolithic disease but more correctly a set of different diseases with different genes that drive growth, proliferation, and metastatic spread. However blocking one genetic upregulated function led to the development of secondary resistance mutations or bypass track mechanism following a clonal selection because of pharmacological exposure. In the near future we will learn how to detect the resistance mechanism and the right therapeutic strategies to allow overcoming resistance mechanisms and thus to transform all oncogene addicted lung cancers as a chronic disease. The employment of immune check-point inhibitors and combinations among immunotherapies probably will bring new hope in non-oncogene addicted or high mutational load lung cancer tumours even if the association with TKIs could be a strategy to overcome resistance mechanism and harness progressive disease in so defined tumour categories.

Finally next-generation sequencing techniques and liquid biopsies will improve the knowledge of tumour biology, i.e. monitoring the resistance mechanisms at the beginning with prevention of tumour progression.

## Conflicts of interest

The authors declare that they have no conflict of interest.

## Authors’ contributions

All authors of this manuscript contributed equally to this work.

## Figures and Tables

**Table 1.** Main clinical studies of ALK TKIs.

Drug	Trial	Phase	Population	Comparator	n	ORR	PFS
Crizotinib	PROFILE 1007	III	Platinum-based pretreated	Pemetrexed/docetaxel	347	65% versus 20%	7.7 versus three months
	PROFILE 1014	III	Naïve	Platinum + pemetrexed	343	74% vs 45%	10.9 versus seven months
Ceritinib	ASCEND 1	I	Advanced malignancies	No	114	58%	seven months
	ASCEND 2	II	Chemo and crizotinib pretreated	No	140	54%	NA
	ASCEND 3	II	Crizo-naive	No	124	79%	11.1 months
	ASCEND 4	III	Treatment naive	Platinum + pemetrexed	NA	NA	NA
	ASCEND 5	III	Platinum based and crizotinib pretreated	Pemetrexed/docetaxel	NA	NA	NA
Alectinib	AF 001JP	I/II	ALK TKI naive	No	46	93.5%	NA
	AF-002JG	I/II	Crizo pretreated	No	47	55%	NA
	NCT01871805	II	Chemotherapy and crizo pretreated	No	NA	NA	NA
	ALTA	III	Naive	Crizotinib	NA	NA	NA
AP26113	NCT01449462	I/II	Crizo pretreated or ALK TKI naive	No	57	69%	10.9 months
	ALTA	II	Crizo pretreated	No	NA	NA	NA

## References

[1] Hirsch FR, Bunn PA (2009). EGFR testing in lung cancer is ready for prime time. Lancet Oncol.

[2] Lynch TJ (2004). Activating mutations in the epidermal growth factor receptor underlying responsiveness of non-small-cell lung cancer to gefitinib. N Engl J Med.

[3] Maemondo M (2010). Gefitinib or chemotherapy for non-small-cell lung cancer with mutated EGFR. N Engl J Med.

[4] Rosell R (2012). Erlotinib versus standard chemotherapy as first-line treatment for European patients with advanced EGFR mutation-positive non-small-cell lung cancer (EURTAC): a multicentre, open-label, randomised phase 3 trial. Lancet Oncol.

[5] Sequist LV (2013). Phase III study of afatinib or cisplatin plus pemetrexed in patients with metastatic lung adenocarcinoma with EGFR mutations. J Clin Oncol.

[6] Yang JC (2015). Afatinib versus cisplatin-based chemotherapy for EGFR mutation-positive lung adenocarcinoma (LUX-Lung 3 and LUX-Lung 6): analysis of overall survival data from two randomised, phase 3 trials. Lancet Oncol.

[7] Kuan FC (2015). Overall survival benefits of first-line EGFR tyrosine kinase inhibitors in EGFR-mutated non-small-cell lung cancers: a systematic review and meta-analysis. Br J Cancer.

[8] Park K (2015). Afatinib (A) vs gefitinib (G) as first-line treatment for patients (pts) with advanced NSCLC harboring activating EGFR mutations: results of the global, randomized, open-label, Phase IIb trial LUX-Lung 7 (LL7). Ann Oncol.

[9] Okabe T (2007). Differential constitutive activation of the epidermal growth factor receptor in non-small cell lung cancer cells bearing EGFR gene mutation and amplification. Cancer Res.

[10] Zhu JQ (2008). Better survival with EGFR exon 19 than exon 21 mutations in gefitinib-treated non-small cell lung cancer patients is due to differential inhibition of downstream signals. Cancer Lett.

[11] Yang JC (2015). Clinical activity of afatinib in patients with advanced non-small-cell lung cancer harbouring uncommon EGFR mutations: a combined post-hoc analysis of LUX-Lung 2, LUX-Lung 3, and LUX-Lung 6. Lancet Oncol.

[12] Arrieta O (2015). The impact of common and rare EGFR mutations in response to EGFR tyrosine kinase inhibitors and platinum-based chemotherapy in patients with non-small cell lung cancer. Lung Cancer.

[13] Riely GJ (2007). Prospective assessment of discontinuation and reinitiation of erlotinib or gefitinib in patients with acquired resistance to erlotinib or gefitinib followed by the addition of everolimus. Clin Cancer Res.

[14] Gandara DR (2014). Acquired resistance to targeted therapies against oncogene-driven non-small-cell lung cancer: approach to subtyping progressive disease and clinical implications. Clin Lung Cancer.

[15] Park K (2016). First-Line Erlotinib Therapy Until and Beyond Response Evaluation Criteria in Solid Tumors Progression in Asian Patients With Epidermal Growth Factor Receptor Mutation-Positive Non-Small-Cell Lung Cancer: The ASPIRATION Study. JAMA Oncol.

[16] Seto T (2014). Erlotinib alone or with bevacizumab as first-line therapy in patients with advanced non-squamous non-small-cell lung cancer harbouring EGFR mutations (JO25567): an open-label, randomised, multicentre, phase 2 study. Lancet Oncol.

[17] Solit DB (2005). Pulsatile administration of the epidermal growth factor receptor inhibitor gefitinib is significantly more effective than continuous dosing for sensitizing tumors to paclitaxel. Clin Cancer Res.

[18] Mok TS (2009). Randomized, placebo-controlled, phase II study of sequential erlotinib and chemotherapy as first-line treatment for advanced non-small-cell lung cancer. J Clin Oncol.

[19] Wu YL (2013). Intercalated combination of chemotherapy and erlotinib for patients with advanced stage non-small-cell lung cancer (FASTACT-2): a randomised, double-blind trial. Lancet Oncol.

[20] Soria JC (2015). Gefitinib plus chemotherapy versus placebo plus chemotherapy in EGFR-mutation-positive non-small-cell lung cancer after progression on first-line gefitinib (IMPRESS): a phase 3 randomised trial. Lancet Oncol.

[21] Schuler M (2016). Afatinib beyond progression in patients with non-small-cell lung cancer following chemotherapy, erlotinib/gefitinib and afatinib: phase III randomized LUX-Lung 5 trial. Ann Oncol.

[22] Ou SH (2012). Second-generation irreversible epidermal growth factor receptor (EGFR) tyrosine kinase inhibitors (TKIs): a better mousetrap? A review of the clinical evidence. Crit Rev Oncol Hematol.

[23] Miller VA (2012). Afatinib versus placebo for patients with advanced, metastatic non-small-cell lung cancer after failure of erlotinib, gefitinib, or both, and one or two lines of chemotherapy (LUX-Lung 1): a phase 2b/3 randomised trial. Lancet Oncol.

[24] Ellis PM (2014). Dacomitinib compared with placebo in pretreated patients with advanced or metastatic non-small-cell lung cancer (NCIC CTG BR.26): a double-blind, randomised, phase 3 trial. Lancet Oncol.

[25] Ramalingam SS (2014). Dacomitinib versus erlotinib in patients with advanced-stage, previously treated non-small-cell lung cancer (ARCHER 1009): a randomised, double-blind, phase 3 trial. Lancet Oncol.

[26] Ramalingam SS (2016). Dacomitinib versus erlotinib in patients with EGFR-mutated advanced nonsmall-cell lung cancer (NSCLC): pooled subset analyses from two randomized trial. Ann Oncol.

[27] Ohashi K (2013). Epidermal growth factor receptor tyrosine kinase inhibitor-resistant disease. J Clin Oncol.

[28] Janne PA (2015). AZD9291 in EGFR inhibitor-resistant non-small-cell lung cancer. N Engl J Med.

[29] Sequist LV (2015). Rociletinib in EGFR-mutated non-small-cell lung cancer. N Engl J Med.

[30] Thress KS (2015). Acquired EGFR C797S mutation mediates resistance to AZD9291 in non-small cell lung cancer harboring EGFR T790M. Nat Med.

[31] Akbay EA (2013). Activation of the PD-1 pathway contributes to immune escape in EGFR-driven lung tumors. Cancer Discov.

[32] D’Incecco A (2015). PD-1 and PD-L1 expression in molecularly selected non-small-cell lung cancer patient. Br J Cancer.

[33] Meyerson M, Gabriel S, Getz G (2010). Advances in understanding cancer genomes through second-generation sequencing. Nat Rev Genet.

[34] Buder A, Tomuta C, Filipits M (2016). The potential of liquid biopsies. Curr Opin Oncol.

[35] Thress KS (2015). EGFR mutation detection in ctDNA from NSCLC patient plasma: A cross-platform comparison of leading technologies to support the clinical development of AZD9291. Lung Cancer.

[36] Oxnard GR (2014). Noninvasive detection of response and resistance in EGFR-mutant lung cancer using quantitative next-generation genotyping of cell-free plasma DNA. Clin Cancer Res.

[37] Karlovich C (2016). Assessment of EGFR Mutation Status in Matched Plasma and Tumor Tissue of NSCLC Patients from a Phase I Study of Rociletinib (CO-1686). Clin Cancer Res.

[38] Soda M (2007). Identification of the transforming EML4-ALK fusion gene in non-small-cell lung cancer. Nature.

[39] Kwak EL (2010). Anaplastic lymphoma kinase inhibition in non-small-cell lung cancer. N Engl J Med.

[40] Koivunen JP (2008). EML4-ALK fusion gene and efficacy of an ALK kinase inhibitor in lung cancer. Clin Cancer Res.

[41] Soda M (2008). A mouse model for EML4-ALK-positive lung cancer. Proc Natl Acad Sci U S A.

[42] Inamura K (2008). EML4-ALK fusion is linked to histological characteristics in a subset of lung cancers. J Thorac Oncol.

[43] Shaw AT (2009). Clinical features and outcome of patients with non-small-cell lung cancer who harbor EML4-ALK. J Clin Oncol.

[44] Birkbak NJ, Hiley CT, Swanton C (2015). Evolutionary precision medicine: a role for repeat epidermal growth factor receptor analysis in ALK-rearranged lung adenocarcinoma?. J Clin Oncol.

[45] Cai W (2015). Intratumoral heterogeneity of ALK-rearranged and ALK/EGFR coaltered lung adenocarcinoma. J Clin Oncol.

[46] Gainor JF (2013). ALK rearrangements are mutually exclusive with mutations in EGFR or KRAS: an analysis of 1,683 patients with non-small cell lung cancer. Clin Cancer Res.

[47] Yang P (2012). Worse disease-free survival in never-smokers with ALK+ lung adenocarcinoma. J Thorac Oncol.

[48] Wu SG (2012). EML4-ALK translocation predicts better outcome in lung adenocarcinoma patients with wild-type EGFR. J Thorac Oncol.

[49] FDA NEWS RELEASE (2011). FDA approves Xalkori with companion diagnostic for a type of late-stage lung cancer. http://www.fda.gov/NewsEvents/Newsroom/.

[50] Takeuchi K (2016). Prospective and clinical validation of ALK immunohistochemistry: results from the phase I/II study of alectinib for ALK-positive lung cancer (AF-001JP study). Ann Oncol.

[51] Wang Y (2015). EML4-ALK Fusion Detected by RT-PCR Confers Similar Response to Crizotinib as Detected by FISH in Patients with Advanced Non-Small-Cell Lung Cancer. J Thorac Oncol.

[52] Leighl NB (2014). Molecular testing for selection of patients with lung cancer for epidermal growth factor receptor and anaplastic lymphoma kinase tyrosine kinase inhibitors: American Society of Clinical Oncology endorsement of the College of American Pathologists/International Association for the study of lung cancer/association for molecular pathology guideline. J Clin Oncol.

[53] Lindeman NI (2013). Molecular testing guideline for selection of lung cancer patients for EGFR and ALK tyrosine kinase inhibitors: guideline from the College of American Pathologists, International Association for the Study of Lung Cancer, and Association for Molecular Pathology. J Thorac Oncol.

[54] Camidge DR (2012). Activity and safety of crizotinib in patients with ALK-positive non-small-cell lung cancer: updated results from a phase 1 study. Lancet Oncol.

[55] Kim DW (2012). Results of a global phase II study with crizotinib in advanced ALK-positive non-small cell lung cancer (NSCLC). J Clin Oncol.

[56] Shaw AT (2013). Crizotinib versus chemotherapy in advanced ALK-positive lung cancer. N Engl J Med.

[57] Solomon BJ (2014). First-line crizotinib versus chemotherapy in ALK-positive lung cancer. N Engl J Med.

[58] Xu CW (2015). Association between EML4-ALK fusion gene and thymidylate synthase mRNA expression in non-small cell lung cancer tissues. Exp Ther Med.

[59] Cappuzzo F (2015). Management of crizotinib therapy for ALK-rearranged non-small cell lung carcinoma: an expert consensus. Lung Cancer.

[60] Camidge DR, Doebele RC (2012). Treating ALK-positive lung cancer--early successes and future challenges. Nat Rev Clin Oncol.

[61] Ignatius Ou SH (2014). Next-generation sequencing reveals a Novel NSCLC ALK F1174V mutation and confirms ALK G1202R mutation confers high-level resistance to alectinib (CH5424802/RO5424802) in ALK-rearranged NSCLC patients who progressed on crizotinib. J Thorac Oncol.

[62] Ou SH (2014). Identification of a novel HIP1-ALK fusion variant in Non-Small-Cell Lung Cancer (NSCLC) and discovery of ALK I1171 (I1171N/S) mutations in two ALK-rearranged NSCLC patients with resistance to Alectinib. J Thorac Oncol.

[63] Doebele RC (2012). Mechanisms of resistance to crizotinib in patients with ALK gene rearranged non-small cell lung cancer. Clin Cancer Res.

[64] Giri S, Patel J K, Mahadevan D (2014). Novel mutations in a patient with ALK-rearranged lung cancer. N Engl J Med.

[65] Kogita A (2014). Hypoxia induces resistance to ALK inhibitors in the H3122 non-small cell lung cancer cell line with an ALK rearrangement via epithelial-mesenchymal transition. Int J Oncol.

[66] Lovly CM (2014). Rationale for co-targeting IGF-1R and ALK in ALK fusion-positive lung cancer. Nat Med.

[67] Anai S (2014). A Case of crizotinib-resistant lung adenocarcinoma harboring a KRAS mutation and an EML4-ALK fusion gene. J Med Cases.

[68] Solomon BJ (2016). Intracranial efficacy of crizotinib versus chemotherapy in patients with advanced ALK-positive non-small-cell lung cancer: results rrom PROFILE 1014. J Clin Oncol.

[69] Ou SH (2014). Clinical benefit of continuing ALK inhibition with crizotinib beyond initial disease progression in patients with advanced ALK-positive NSCLC. Ann Oncol.

[70] Chiari R (2015). Clinical impact of sequential treatment with ALK-TKIs in patients with advanced ALK-positive non-small cell lung cancer: Results of a multicenter analysis. Lung Cancer.

[71] Costa DB (2011). CSF concentration of the anaplastic lymphoma kinase inhibitor crizotinib. J Clin Oncol.

[72] Johung KL (2016). Extended Survival and Prognostic Factors for Patients With ALK-Rearranged Non-Small-Cell Lung Cancer and Brain Metastasis. J Clin Oncol.

[73] Baik CS, Chamberlain MC, Chow LQ (2015). Targeted Therapy for Brain Metastases in EGFR-Mutated and ALK-Rearranged Non-Small-Cell Lung Cancer. J Thorac Oncol.

[74] Zhang I (2015). Targeting brain metastases in ALK-rearranged non-small-cell lung cancer. Lancet Oncol.

[75] Friboulet L (2014). The ALK inhibitor ceritinib overcomes crizotinib resistance in non-small cell lung cancer. Cancer Discov.

[76] Shaw AT (2014). Ceritinib in ALK-rearranged non-small-cell lung cancer. N Engl J Med.

[77] Kim DW (2014). Ceritinib in advanced anaplastic lymphoma kinase (ALK)-rearranged (ALK+) non-small cell lung cancer (NSCLC): Results of the ASCEND-1 trial. J Clin Oncol.

[78] Seto T (2013). CH5424802 (RO5424802) for patients with ALK-rearranged advanced non-small-cell lung cancer (AF-001JP study): a single-arm, open-label, phase 1-2 study. Lancet Oncol.

[79] Gadgeel SM (2014). Safety and activity of alectinib against systemic disease and brain metastases in patients with crizotinib-resistant ALK-rearranged non-small-cell lung cancer (AF-002JG): results from the dose-finding portion of a phase 1/2 study. Lancet Oncol.

[80] Ou SH (2016). Alectinib in Crizotinib-Refractory ALK-Rearranged Non-Small-Cell Lung Cancer: A Phase II Global Study. J Clin Oncol.

[81] Shaw AT (2016). Alectinib in ALK-positive, crizotinib-resistant, non-small-cell lung cancer: a single-group, multicentre, phase 2 trial. Lancet Oncol.

[82] No authors listed (2016). Alectinib Approved for ALK+ Lung Cancer. Cancer Discov.

[83] Squillace RM (2013). AP26113 possesses pan-inhibitory activity versus crizotinib-resistant ALK mutants and oncogenic ROS1 fusions. Cancer Res.

[84] Gettinger SN (2014). Updated efficacy and safety of the ALK inhibitor AP26113 in patients (pts) with advanced malignancies, including ALK+ non-small cell lung cancer (NSCLC). J Clin Oncol.

[85] Shaw AT (2016). Resensitization to Crizotinib by the Lorlatinib ALK Resistance Mutation L1198F. N Engl J Med.

[86] Shaw AT (2015). Clinical activity and safety of PF-06463922 from a dose escalation study in patients with advanced ALK+ or ROS1+ NSCLC. J Clin Oncol.

[87] Toyokawa G, Seto T (2015). Updated Evidence on the Mechanisms of Resistance to ALK Inhibitors and Strategies to Overcome Such Resistance: Clinical and Preclinical Data. Oncol Res Treat.

[88] Hoffmann-La Roche ALEX Study: A Randomized, Phase III Study Comparing Alectinib With Crizotinib in Treatment-Naive Anaplastic Lymphoma Kinase-Positive Advanced Non-Small Cell Lung Cancer Participants. ClinicalTrials.gov, Identifier: NCT02075840.

[89] Govindan R (2015). ALCHEMIST Trials: A Golden Opportunity to Transform Outcomes in Early-Stage Non-Small Cell Lung Cancer. Clin Cancer Res.

[90] Davies H (2002). Mutations of the BRAF gene in human cancer. Nature.

[91] Chapman PB (2011). Improved survival with vemurafenib in melanoma with BRAF V600E mutation. N Engl J Med.

[92] Peters S, Michielin O, Zimmermann S (2013). Dramatic response induced by vemurafenib in a BRAF V600E-mutated lung adenocarcinoma. J Clin Oncol.

[93] Robinson SD (2014). BRAF V600E-mutated lung adenocarcinoma with metastases to the brain responding to treatment with vemurafenib. Lung Cancer.

[94] Planchard D (2016). Dabrafenib in patients with BRAF(V600E)-positive advanced non-small-cell lung cancer: a single-arm, multicentre, open-label, phase 2 trial. Lancet Oncol.

[95] Gandara DR (2013). Oral MEK1/MEK2 inhibitor trametinib (GSK1120212) in combination with docetaxel in KRAS-mutant and wild-type (WT) advanced non-small cell lung cancer (NSCLC): A phase I/Ib trial. J Clin Oncol.

[96] Pietsch T (1998). Expression of the c-Kit receptor and its ligand SCF in non-small-cell lung carcinomas. Int J Cancer.

[97] Lu HY (2012). Expression and mutation of the c-kit gene and correlation with prognosis of small cell lung cancer. Oncol Lett.

[98] Krug LM (2005). Randomized phase II study of weekly docetaxel plus trastuzumab versus weekly paclitaxel plus trastuzumab in patients with previously untreated advanced nonsmall cell lung carcinoma. Cancer.

[99] Gatzemeier U (2004). Randomized phase II trial of gemcitabine-cisplatin with or without trastuzumab in HER2-positive non-small-cell lung cancer. Ann Oncol.

[100] De Greve J (2012). Clinical activity of afatinib (BIBW 2992) in patients with lung adenocarcinoma with mutations in the kinase domain of HER2/neu. Lung Cancer.

[101] Garrido-Castro A C, Felip E (2013). HER2 driven non-small cell lung cancer (NSCLC): potential therapeutic approaches. Transl Lung Cancer Res.

[102] Harada D (2012). JAK2-related pathway induces acquired erlotinib resistance in lung cancer cells harboring an epidermal growth factor receptor-activating mutation. Cancer Sci.

[103] Looyenga BD (2012). STAT3 is activated by JAK2 independent of key oncogenic driver mutations in non-small cell lung carcinoma. PLoS One.

[104] Dearden S (2013). Mutation incidence and coincidence in non small-cell lung cancer: meta-analyses by ethnicity and histology (mutMap). Ann Oncol.

[105] Janne PA (2013). Selumetinib plus docetaxel for KRAS-mutant advanced non-small-cell lung cancer: a randomised, multicentre, placebo-controlled, phase 2 study. Lancet Oncol.

[106] Mellema WW (2015). Comparison of clinical outcome after first-line platinum-based chemotherapy in different types of KRAS mutated advanced non-small-cell lung cancer. Lung Cancer.

[107] Rizvi NA (2015). Cancer immunology. Mutational landscape determines sensitivity to PD-1 blockade in non-small cell lung cancer. Science.

[108] Menis J, Giaj Levra M, Novello S (2013). MET inhibition in lung cancer. Transl Lung Cancer Res.

[109] Scagliotti G (2015). Phase III Multinational, Randomized, Double-Blind, Placebo-Controlled Study of Tivantinib (ARQ 197) Plus Erlotinib Versus Erlotinib Alone in Previously Treated Patients With Locally Advanced or Metastatic Nonsquamous Non-Small-Cell Lung Cancer. J Clin Oncol.

[110] Spigel DR (2013). Randomized phase II trial of Onartuzumab in combination with erlotinib in patients with advanced non-small-cell lung cancer. J Clin Oncol.

[111] Spigel DR (2014). Onartuzumab plus erlotinib versus erlotinib in previously treated stage IIIb or IV NSCLC: Results from the pivotal phase III randomized, multicenter, placebo-controlled METLung (OAM4971g) global trial. J Clin Oncol.

[112] Rolfo C (2015). Onartuzumab in lung cancer: the fall of Icarus?. Expert Rev Anticancer Ther.

[113] Camidge DR (2014). Efficacy and safety of crizotinib in patients with advanced c-MET-amplified non-small cell lung cancer (NSCLC). J Clin Oncol.

[114] Paik PK (2015). Response to crizotinib and cabozantinib in stage IV lung adenocarcinoma patients with mutations that cause MET exon 14 skipping. J Clin Oncol.

[115] Sequist LV (2014). Phase I study of BGJ398, a selective pan-FGFR inhibitor in genetically preselected advanced solid tumors. Cancer Res.

[116] Oxnard G R, Binder A, Janne PA (2013). New targetable oncogenes in non-small-cell lung cancers. J Clin Oncol.

[117] Drilon A (2013). Response to Cabozantinib in patients with RET fusion-positive lung adenocarcinomas. Cancer Discov.

[118] Falchook GS (2014). Effect of the RET Inhibitor Vandetanib in a Patient With RET Fusion-Positive Metastatic Non-Small-Cell Lung Cancer. J Clin Oncol.

[119] Lipson D (2012). Identification of new ALK and RET gene fusions from colorectal and lung cancer biopsies. Nat Med.

[120] Kodama T (2014). Alectinib shows potent antitumor activity against RET-rearranged non-small cell lung cancer. Mol Cancer Ther.

[121] Shaw AT (2014). Crizotinib in ROS1-rearranged non-small-cell lung cancer. N Engl J Med.

[122] Katayama R (2015). Cabozantinib overcomes crizotinib resistance in ROS1 fusion-positive cancer. Clin Cancer Res.

[123] Zou HY (2015). PF-06463922 is a potent and selective next-generation ROS1/ALK inhibitor capable of blocking crizotinib-resistant ROS1 mutations. Proc Natl Acad Sci U S A.

[124] Garon EB (2015). Pembrolizumab for the treatment of non-small-cell lung cancer. N Engl J Med.

[125] Borghaei H (2015). Nivolumab versus docetaxel in advanced nonsquamous non-small-cell lung cancer. N Engl J Med.

[126] Brahmer J (2015). Nivolumab versus Docetaxel in Advanced Squamous-Cell Non-Small-Cell Lung Cancer. N Engl J Med.

[127] Spira AI (2015). Efficacy, safety and predictive biomarker results from a randomized phase II study comparing MPDL3280A vs docetaxel in 2L/3L NSCLC (POPLAR). J Clin Oncol.

[128] Schwaederle M (2016). Detection rate of actionable mutations in diverse cancers using a biopsy-free (blood) circulating tumor cell DNA assay. Oncotarget.

[129] Le Tourneau C (2015). Molecularly targeted therapy based on tumour molecular profiling versus conventional therapy for advanced cancer (SHIVA): a multicentre, open-label, proof-of-concept, randomised, controlled phase 2 trial. Lancet Oncol.

[130] Barlesi F (2016). Routine molecular profiling of patients with advanced non-small-cell lung cancer: results of a 1-year nationwide programme of the French Cooperative Thoracic Intergroup (IFCT). Lancet.

